# Safety and efficacy of camrelizumab combined with radiotherapy as neoadjuvant therapy for locally advanced esophageal squamous cell carcinoma: a prospective single-arm phase II clinical trial protocol

**DOI:** 10.1186/s13063-023-07534-3

**Published:** 2023-08-25

**Authors:** Maohui Chen, Yizhou Huang, Shuliang Zhang, Taidui Zeng, Guanglei Huang, Chun Chen, Bin Zheng

**Affiliations:** 1 Department of Thoracic Surgery, Fujian Medical University Union Hospital, 29 Xinquan Road, Fuzhou, Fujian China; 2 Key Laboratory of Cardio-Thoracic Surgery (Fujian Medical University), Fujian Province University, Fuzhou, Fujian China; 3 National Key Clinical Specialty of Thoracic Surgery, Fuzhou, China

**Keywords:** Esophageal cancer, Neoadjuvant therapy, Immunotherapy, Radiotherapy, Prospective research

## Abstract

**Background:**

Neoadjuvant chemoradiotherapy followed by esophagectomy is the standard of care for locally advanced esophageal squamous cell carcinoma (ESCC). However, approximately 30% of patients still develop distant metastases and have a high incidence of treatment-related adverse events. Immunotherapy, as a new modality for anti-cancer treatment, has shown promising clinical benefits for patients with ESCC. The synergistic effects of immunotherapy and radiotherapy make their combination promising as neoadjuvant treatment for locally advanced ESCC.

**Methods:**

All participants who meet the inclusion criteria will be enrolled after signing the informed consent form. Patients with thoracic segment esophageal cancer with clinical stage T2–3 N0 M0 or T2–3 N + M0 will be included. A total of 25 patients are to be recruited for the study. Twelve patients will be recruited in phase I, with at least two achieving major pathological response (MPR) before entering phase II. They will be treated with radical surgery within 4–8 weeks after the completion of two cycles of neoadjuvant radiotherapy in combination with camrelizumab according to the study schedule. The primary endpoint is the major pathological remission rate of all per-protocol patients. The secondary endpoints are the R0 resection rate, pathological complete remission rate, and adverse events. The interim analysis will be conducted after 12 patients have been enrolled. The trials will be terminated when more than two treatment-related deaths occur or fewer than five patients have major pathological remission.

**Discussion:**

We designed this prospective single-arm phase II clinical study to evaluate the combination of camrelizumab and standard radiotherapy as preoperative neoadjuvant therapy for patients with resectable ESCC as part of the quest for better treatment options for patients with locally advanced ESCC.

**Trial registration:**

This trial protocol has been registered on the NIH Clinical Trials database (www.clinicaltrials.gov/, NCT05176002. Registered on 2022/01/04). The posted information will be updated as needed to reflect protocol amendments and study progress.

## Background

Esophageal cancer is the seventh most common cancer and the sixth most common cause of cancer-related death globally [1]. Its most prevalent pathological type is esophageal squamous cell carcinoma (ESCC), which accounts for more than 90% of cases globally, and China is among the regions with the highest risk [2]. The National Comprehensive Cancer Network and Chinese Society of Clinical Oncology guidelines recommend neoadjuvant radiotherapy followed by esophagectomy as the standard of care for locally advanced ESCC [3]. However, the CROSS and NEOCRTEC5010 studies reported a high incidence of distant metastases after neoadjuvant chemoradiotherapy at 39% and 23.9%, respectively [4, 5]. Therefore, a more effective systemic antitumor regimen is the next priority for the comprehensive treatment of ESCC.

In recent years, immunotherapy has demonstrated clinical benefits as a new treatment regimen for patients with locally advanced and metastatic ESCCs [6, 7]. The PALACE-1 study reported that 20 cases of locally advanced ESCC were treated with neoadjuvant chemoradiotherapy combined with immunotherapy, and surgery was successful for 18 cases; only one case of tumor progression and another case of death during treatment were reported. The pathological complete remission (pCR) rate was 55.6%, which was higher than those reported by the NEOCRTEC5010 and CROSS studies. However, a higher prevalence of treatment-related adverse events (AEs) was also reported, with grade III or higher AEs observed in 65% of patients [8]. Li et al. showed that 60 patients with locally advanced ESCC received neoadjuvant camrelizumab immunotherapy in combination with chemotherapy, and 51 received neoadjuvant therapy and underwent surgery successfully. The pCR rate was 39.2%, and the most common treatment-related AE was leukopenia (86.7%). Thirty-four patients (56.7%) had grade 3 or worse AEs [9]. The treatment-related AEs predominantly involved the hematologic and digestive systems, both of which are commonly involved in AEs of chemotherapy [8, 9].

Some studies have suggested that conventional radiation therapy is effective by promoting systemic anti-tumor immunity, thereby reducing the risk of recurrence and improving patient survival [10]. For other types of malignancies, immunotherapy combined with radiotherapy has demonstrated significantly better efficacy with an acceptable incidence of AEs [11, 12]. Relevant preclinical studies have shown that the effects of radiotherapy and immunotherapy are synergistic, rather than simply cumulative [13, 14]. Immunotherapy has been reported to increase radiosensitivity in patients with ESCC, resulting in effective control of local lesions and distant micrometastases, while radiotherapy can enhance the effect of immunotherapeutic agents by upregulating PD-L1 expression in the tumor microenvironment [15, 16]. To further evaluate the prospects of immunotherapy combined with radiotherapy as neoadjuvant treatment for patients with locally advanced ESCC, we designed this single-center, single-arm, prospective phase 2 clinical study as part of the quest for practical, novel therapeutic options for neoadjuvant locally advanced ESCC.

## Methods/design

### Study objectives

The primary objective of this study is to assess the safety of camrelizumab combined with standard radiotherapy as preoperative neoadjuvant therapy for patients with locally advanced ESCC, and the secondary objective is to assess the efficacy of the neoadjuvant therapy. The present study also aims to explore the biomarkers and the quality of life in patients with locally advanced ESCC.

### Study design

This prospective study is a single-center, single-arm, prospective phase II clinical study. All participants who meet the inclusion criteria will be enrolled after signing the informed consent form. A total of 25 patients will need to be recruited for the study. Twelve patients will be enrolled in phase I, with at least two patients achieving MPR before entering phase II. The second phase will enroll 13 patients. They will be treated with radical surgery within 4–8 weeks after the completion of two cycles of neoadjuvant radiotherapy in combination with camrelizumab according to the study schedule. The schedule of enrollment, interventions, and assessments is shown in Table 1. If the total number of MPR patients is greater than 7, the results of this study will be considered satisfactory.

**Table 1:** Patient assessment schedule

	**Study period**									
**Timepoint**	**Enrolment**	**Combined therapy** **Camrelizumab + Radiation**		**Surgery**	***Follow-up after surgery***					**Close-out**
	***0***	**Cycle 1**	**Cycle 2**	***1 week***	***4 weeks***	***12 weeks***		***Every 6 months***		***t***_***x***_
**Enrolment:**										
**Eligibility screen**	X									
**Informed consent**	X									
***Assessments:***										
**Baseline patient characteristics**	X									
**Baseline tumor characteristics**	X									
***Laboratory examination***	X	X	X							
***Tumor response evaluation***		X	X							
***Postoperative outcome data***				X						
***pathological assessment***				X						
***Adverse events assessment***		X	X	X	X		X		X	
***Quality of life measure***					X		X		X	X
***Survival***					X		X		X	X

### Study setting 

All participants will be recruited from the Department of Thoracic Surgery, Fujian Medical University Union Hospital, from September 2021 to September 2023.

### Study endpoints

The primary endpoint is the major pathological remission (MPR) rate in all per-protocol patients. MPR is defined as the remainder of ≤ 10% of viable tumor cells on postoperative pathological examination, and pCR is defined as tumor regression without a residual tumor on pathological examination [17, 18]. The secondary endpoints are the R0 resection rate, pCR rate, and AEs. AEs, including those during neoadjuvant treatment and surgical morbidity and mortality, will be recorded.

### Inclusion criteria

The inclusion criteria are as follows: (a) histologically-confirmed squamous cell carcinoma; (b) primary lesions located in the thoracic esophagus; (c) clinical stages of T2–3 N0 M0 or T2–3 N + M0, based on the 8th UICC-TNM classification; (d) having not received neoadjuvant therapy; (e) 18–75 years; (f) ECOG performance status of 0 or 1; (g) no prior chemotherapy, radiotherapy, or immunotherapy for any cancers; (h) adequate organ function; (i) expectation of R0 resection; and (j) provision of written informed consent.

### Exclusion criteria

The exclusion criteria are as follows: (a) corticosteroid treatment (equivalent to prednisone > 10 mg/day) within 14 days before the first day of drug administration; (b) acquired immunodeficiency syndrome or active hepatitis B (DNA ≥ 104 copies/ml) or C (RNA ≥ 103 copies/ml) viral infections; (c) history of pneumonitis or interstitial lung disease with clinical evidence, such as interstitial pneumonia and pulmonary fibrosis features on baseline CT scans; (d) known or concurrent bleeding disorders or other uncontrolled diseases contraindicating surgical treatment; (e) physical examination or clinical trial findings that can interfere with the results or put the patient at increased risk for treatment complications; (f) comorbidities, including chronic pulmonary disease, poorly controlled hypertension, unstable angina, myocardial infarction within 6 months, and unstable mental disorders requiring therapy; (g) allergy to drugs used in the study; (i) participation in other clinical trials within 30 days before enrollment; and (j) ineligibility for participation based on the decision of investigators.

Patients to be considered ineligible for participation by the investigators include the following: (a) patients with second primary malignant disease; (b) patients with hypersensitivity to the investigational drug camrelizumab and chemotherapeutic agents; (c) patients with concurrent autoimmune disease or history of chronic autoimmune disease; (d) participants of other clinical trials within the prior 4 weeks; (e) patients with a history of systemic therapy with immunomodulatory agents for antitumor indications within the prior 2 weeks; (f) patients with an active autoimmune disease requiring systemic therapy within 2 years before the first dose; (g) patients with known allogeneic organ transplantation (except corneal transplantation) or allogeneic hematopoietic stem cell transplantation; (h) patients with severe or uncontrollable systemic disease; (i) patients with medical history or evidence of disease that may interfere with the results of the trial or prevent participation in the study over its entire duration, abnormal treatment outcomes or laboratory test results, or other potential risks deemed by the investigators to be inappropriate for participation in this study.

### Interventions

Patients will receive two cycles of neoadjuvant radiotherapy combined with camrelizumab according to the study schedule, and they will undergo radical surgery within 4–8 weeks after neoadjuvant therapy. The details of the neoadjuvant treatment are as follows.

#### Immunotherapy

The patients will receive two courses of preoperative camrelizumab (200 mg/day, day 1) repeated every 3 weeks.

#### Radiotherapy

Three-dimensional conformal or intensity-modulated radiotherapy will be administered at a total radiation dose of 4140 cGy (180 cGy at a time for five times a week). The targeted areas are as follows: CTV will include 3 cm above and below the preoperative primary lesion and the regional lymphatic drainage area, where the metastatic lymph nodes were located; and PTV will include the area targeted by CTV extended by 5 mm.

#### Surgery

The patients will be re-evaluated by the investigators according to the Response Evaluation Criteria for Solid Tumors version 1.1 approximately 4–8 weeks after neoadjuvant treatment. Esophagectomy will be performed if there is no evidence of metastatic disease. Patients will undergo robotic minimally invasive esophagectomy, minimally invasive esophagectomy, or open right thoracotomy esophagectomy with 2- or 3-field lymph node dissection. Transhiatal or left thoracotomy esophagectomy is not acceptable due to the limited capacity for upper mediastinum lymph node dissection. Surgery-related complications will be recorded on the case report form for up to 90 days after surgery.

### Adverse events record and safety assessment

AEs will be recorded and graded according to the Common Terminology Criteria for Adverse Event (CTCAE) version 5.0 [19]. Throughout the trial, all potential AEs will be monitored under the supervision of the Ethics Review Committee of Fujian Medical University Union Hospital. Patients can withdraw from the trial at any time if they cannot tolerate the treatment.

### Interim analysis and monitoring

We plan to conduct one interim analysis. The interim analysis will be conducted after 12 patients have been enrolled. The Lan-DeMets method with the O’Brien and Fleming type alpha spending function will be used to adjust the multiplicity of the interim analysis [20]. Trials will be terminated when more than two treatment-related deaths occur or there are fewer than two patients with MPR based on the analysis.

### Statistical analysis

After obtaining written informed consent, the medical records of participants will be continually reviewed and checked by the study staff, and relevant data will be collected and recorded. All statistical analyses will be performed using SPSS 22.0 (or higher), and all statistical tests will be one-sided. 0.05 hypothesis of superiority tests, and 95% confidence intervals and *p*-values will be provided for group comparisons. Unless otherwise stated, the data will be summarized using mean ± standard deviation or median (minimum, maximum). Countable data will be summarized using frequency (percentage).

### Patient and public involvement

Neither patients nor public will be involved in the design, recruitment, outcome measures, and conduct of the study. Trial results will be disseminated via peer-reviewed scientific journals and conference presentations rather than specifically notified to a single patient.

## Discussion

This is a single-arm, single-center prospective phase II study to evaluate the safety and efficacy of camrelizumab immunotherapy combined with radiotherapy as preoperative neoadjuvant therapy for patients with locally advanced ESCC as part of the exploration of better treatment options for locally advanced resectable ESCC.

The CROSS and NEOCRTEC5010 studies demonstrated the benefits of neoadjuvant radiotherapy for locally advanced ESCC [4, 5]. The NEOCRTEC5010 trial was a phase III randomized trial involving Chinese ESCC patients, who were divided into neoadjuvant chemoradiotherapy and surgery-only groups. The pCR rate for the neoadjuvant chemoradiotherapy group was as high as 43.2%, with a median survival duration of 100.1 months, compared with 41.7 months for the surgery-only group (*p* < 0.001). However, the superimposed toxicity of chemotherapy and radiotherapy may lead to serious acute AEs during radiotherapy, such as severe bone marrow inhibition, pneumonia, and esophageal perforation, which may limit the clinical application of neoadjuvant chemoradiotherapy [21, 22]. Moreover, neoadjuvant chemoradiotherapy may result in adhesion and edema of peritumor tissues and further increase the difficulty of surgical procedures and the incidence of perioperative complications [23, 24].

Immune checkpoint inhibitors have been proven to be effective for the perioperative treatment of esophageal cancers [25, 26]. The available clinical data on immune checkpoint inhibitors and their combinations with radiotherapy for the preoperative treatment of esophageal cancer are promising [27, 28]. However, some studies have suggested that immunotherapy combined with radiotherapy is associated with a higher incidence of AEs. A phase 2 clinical trial investigating neoadjuvant radiotherapy combined with atezolizumab for resectable esophageal adenocarcinoma showed a pCR rate of 25%, but grade III or higher AEs were observed in 42.5% of the patients [29]. The PALACE-1 study also demonstrated a high incidence of AEs, with grade III or higher AEs in up to 65% of patients and one death reported during treatment [8].

Several studies have demonstrated the efficacy of the preoperative combination of immunotherapy and chemotherapy in the treatment of esophageal cancer. Jun et al. conducted a study of neoadjuvant treatment of locally advanced ESCC with camrelizumab combined with chemotherapy. Of the 60 patients recruited, 51 eventually underwent surgery with a pCR rate of 39.2%. However, the study also reported that 39 (65.0%) patients experienced grade 3 or more severe AEs, with the most common being leukopenia (50.0%) [9]. The combination of immunotherapy and chemotherapy has an acceptable pathological remission rate. However, the associated chemotherapy-related AEs are still of concern, given that they are difficult to avoid with the current clinical application of this regimen. This is the rationale for exploring the new adjuvant strategy of combining immunotherapy and radiotherapy to possibly replace the combination of chemotherapy and immunotherapy. The combination of immunotherapy and radiotherapy has potent systemic antitumor effects and is expected to provide better survival benefits with fewer treatment-related AEs.

Previous clinical studies have demonstrated several synergistic effects of immunotherapy and radiotherapy [13]. It was demonstrated that radiotherapy can enhance the effect of immunotherapeutic drugs by upregulating PD-L1 expression in the tumor microenvironment, while immune drugs can increase radiosensitivity in ESCC patients and facilitate the effective control of local lesions and distant micrometastases [30, 31]. In clinical practice, it has been observed that RT, in addition to its direct local tumor control, induces an immune response, the so-called non-local effect to control distant metastases. This effect can have cytotoxic effects on tumor cells far from the irradiated area, which is expected to eliminate metastases after combination therapy [15]. For other types of malignant tumors, immunotherapy combined with radiotherapy has shown remarkable efficacy. The MDACC trial showed that combining radiotherapy and Pembrolizumab immunotherapy significantly increased response and outcomes in patients with metastatic non-small cell lung cancer [32]. Another study also reported that the combination of immunotherapy and radiotherapy improved overall survival in patients with melanoma and brain metastases [33]. All the above studies have suggested the potential of the combination of immunotherapy and radiotherapy as a therapeutic modality for comprehensive tumor treatment. Consequently, an investigation of the efficacy of immunotherapy combined with radiotherapy for the treatment of locally advanced esophageal cancer is worthwhile.

In conclusion, the ideal neoadjuvant therapy is associated with high pCR rates, long-term survival, and fewer treatment-related AEs. As part of the exploration of better neoadjuvant treatment options for esophageal squamous cancer, we designed this prospective single-arm phase II clinical study to evaluate the combination of a PD-1 monoclonal antibody and standard radiotherapy for preoperative neoadjuvant therapy for patients with resectable esophageal squamous cancer to provide practical and better treatment options for ESCCs.

## Trial status

Version 1.0, September 7, 2021.

Start of inclusion: February 15, 2022.

Finish date of recruitment: December 15, 2023.

## Data Availability

The dataset used and analyzed in this study will be available from the corresponding author upon reasonable request.

## Abbreviations

AEs: Adverse events
CSCO: Chinese Society of Clinical Oncology
EC: Esophageal cancer
ESCC: Esophageal squamous cell carcinoma
MPR: Major pathological remission
NCCN: National Comprehensive Cancer Network
pCR: Pathological complete response
